# 

**Published:** 1872-12

**Authors:** 


☞ RENEW YOUR SUBSCRIPTIONS. ☜

**PARTICULAR NOTICE.**

☞ *THE JOURNAL will not be sent, after the present number, to any who have not renewed their subscriptions.*

☞ **Remit $3 for the new volume, commencing January, 1873.**

☞ **Complete Sets for last year can be furnished for $3.**

